# Impact of Sinus Rhythm Restoration on Mitral Regurgitation in Patients With Atrial Fibrillation: A Comparative Echocardiographic Study

**DOI:** 10.7759/cureus.69010

**Published:** 2024-09-09

**Authors:** Sameer M Mtour, Ali Mtour, Yumna Njoum, Lila H Abu-Hilal, Farah Jabbarin, Bilal Adwan, Monther Nassar, Raghad Dannan, Isaac Alsallamin

**Affiliations:** 1 Cardiology, Al-Makassed Hospital, Jerusalem, PSE; 2 Medicine and Surgery, Al-Quds University, Jerusalem, PSE; 3 Medicine, Al-Quds University, Jerusalem, PSE; 4 General Practice, Al-Quds University, Jerusalem, PSE; 5 Internal Medicine, University Hospitals Cleveland Medical Center, Cleveland, USA; 6 Clinical Medicine, Northeast Ohio Medical University, Cleveland, USA; 7 Internal Medicine, St. Vincent Charity Medical Center, Cleveland, USA; 8 Internal Medicine, Case Western Reserve University School of Medicine, Cleveland, USA

**Keywords:** atrial fibrillation (afib), cardioversion, echocardiogram, mitral valve, mitral valve dysfunction, mitral valve insufficiency, palestine, sinus rythm

## Abstract

Introduction: Mitral regurgitation (MR) is a common valvular abnormality that can be exacerbated by atrial fibrillation (AF). Mitral regurgitation is classified based on mitral leaflet motion and can be either primary (organic) or secondary (functional). This study investigates the relationship between AF and functional MR, specifically assessing whether cardioversion to sinus rhythm influences MR severity and echocardiographic indices.

Methods: This retrospective cohort study included 417 patients with AF and significant MR (Grade III or IV) who were hospitalized for cardioversion. Patients underwent echocardiography during AF and again within six months after successful cardioversion. The primary outcome was the change in MR severity post cardioversion. Secondary outcomes included changes in left atrial size, left ventricular ejection fraction, and mitral valve anatomy. Statistical analysis involved chi-square tests for categorical variables, Student's t-tests for continuous variables, and multivariate logistic regression to assess the impact of sinus rhythm restoration on MR severity.

Results: Of the 417 patients, 61% (n = 254) returned to sinus rhythm after cardioversion. Among these, 28.4% (n = 72) showed a two-grade or greater improvement in MR severity. Patients who returned to sinus rhythm also exhibited significant reductions in left ventricular and left atrial dimensions and improved transvalvular gradients. The multivariate analysis indicated that conversion to sinus rhythm was independently associated with MR improvement.

Conclusions: Restoration of sinus rhythm is associated with significant improvement in functional MR, potentially reducing the need for surgical intervention. These findings suggest that rhythm control should be considered in managing patients with AF and significant MR, as it may lead to improved cardiac function and better clinical outcomes. Further large-scale prospective studies are needed to determine the most effective management strategy for functional MR in the context of AF.

## Introduction

The mitral valve is a complex structure that relies on the coordinated function of its components, including the leaflets, chordae tendineae, papillary muscles, left ventricle, and mitral annulus, to maintain normal operation [1, 2]. Disruption of any part of this system can lead to mitral regurgitation (MR), a common valvular abnormality that affects over 2% of the global population, with increasing prevalence with age [1]. Mitral regurgitation is the most frequent type of valvular abnormality and can be classified based on mitral leaflet motion according to Carpentier's classification into normal motion (Type I), excessive motion (Type II), or restrictive motion (Type III) [1, 2].

Mitral regurgitation can also be categorized as primary (organic) MR involving mitral leaflet disease or secondary (functional) MR, which affects the leaflets indirectly. Organic Type II MR is often associated with myxomatous degeneration, while secondary Type II MR typically results from ruptured chordae or papillary muscles. Rheumatic valve disease is a common cause of organic Type III MR, whereas functional MR can result from dilated cardiomyopathy. Mitral regurgitation with normal leaflet motion is relatively rare and is almost exclusively the result of organic leaflet disease leading to annular dilation [1,3,4].

Carpentier's analysis of 551 surgical cases of MR found that over 98% of patients with Type I MR had valvular dysfunction due to annular dilation, primarily caused by rheumatic disease. Although organic Type I MR is uncommon, it can arise from extensive mitral annular calcification, endocarditis-related perforation, or a congenitally cleft mitral leaflet. The development of functional Type I MR, which is less well understood, has been linked to atrial fibrillation (AF). Atrial fibrillation can cause atrial remodeling and mitral annular dilation, resulting in a loss of synchronous atrial mechanical activity, reduced ventricular filling time, and decreased cardiac output. When combined with a persistently elevated ventricular rate, these factors can impair coronary flow, reduce myocardial contractility, increase left ventricular dimension, and exacerbate MR [5]. According to the American Society of Echocardiography (ASE) classification, MR severity is categorized as follows: a regurgitant fraction of less than 30% indicates mild MR (Grade I), 30% to 39% indicates mild-to-moderate MR (Grade II), 40% to 49% indicates moderate-to-severe MR (Grade III), and 50% or greater indicates severe MR (Grade IV) [6].

Atrial fibrillation is a condition that can lead to functional Type I MR through atrial remodeling, which results in mitral annular dilation. As the most prevalent arrhythmia, AF is commonly observed in patients aged 80 and older, affecting 10%-12% of this age group. The condition arises from both structural and electrical changes in atrial tissue. A major factor is the formation of fibrotic tissue and the disruption of electrical signals. This fibrosis can be either reactive, involving the replacement of damaged cells, or infiltrative, involving the accumulation of glycosphingolipids or insoluble proteins in the interstitial space [7-9].

Treatment options for AF include cardioversion, rate control, rhythm control, and ablation therapy. Given the association between atrial remodeling and MR, managing AF could potentially influence the extent of MR. Recent studies suggest that rate control can be as effective as rhythm control for AF, though its impact on mitral regurgitation is not well documented. Cardioversion remains a primary treatment for AF, but there is limited research on its effectiveness in reducing the severity of MR [9, 10].

The hemodynamic impact of AF includes the loss of coordinated atrial contraction and reduced ventricular filling time, which can diminish cardiac output. Persistent rapid ventricular rates during AF can further compromise coronary flow, decrease myocardial contractility, increase left ventricular dimensions, enlarge the mitral valve annulus, and worsen MR [10-14].

In this study, we focused on patients with significant MR and AF who underwent cardioversion at our institution. Our primary objective was to investigate the potential association between AF and MR, specifically to determine if AF contributes to at least moderate MR severity. By comparing mitral valve function before and after AF cardioversion, this study aims to identify the impact of AF on valve dysfunction and assess its potential reversibility. We also aim to evaluate various echocardiographic indices in patients who achieved sinus rhythm and to assess the correlation between these indices and improvement in MR severity. We aim to elucidate specific correlations between these indices and MR severity improvement through a comprehensive analysis of echocardiographic indices before and after cardioversion. Conducting this study in Palestine provides valuable local data that can inform clinical practice and management decisions for patients with significant MR and AF in our region.

## Materials and methods

This retrospective cohort study included patients with AF who were hospitalized for cardioversion at our hospital. Inclusion criteria were patients aged 18 years or older with significant MR demonstrated on echocardiography during AF who underwent successful cardioversion and had follow-up echocardiography within six months. Significant MR was defined as Grade III or Grade IV according to the ASE classification. Exclusion criteria included a history of mitral valve surgery, other significant valvular disease, evidence of primary leaflet involvement (e.g., prior endocarditis, rheumatic valve disease, congenital anomaly, or significant mitral annular calcification), and inadequate echocardiographic data. Ethical approval by the Institutional Review Board was waived due to the retrospective nature of the study design.

Data were collected retrospectively from electronic medical records. All patients underwent echocardiography during AF, which confirmed significant MR. Patients were then followed up with an additional echocardiography within six months after successful cardioversion. Echocardiographic indices from the initial and follow-up tests were recorded and compared. The primary outcome was the change in MR severity after cardioversion. Secondary outcomes included changes in other echocardiographic indices, such as left atrial size, left ventricular ejection fraction, and mitral valve anatomy.

Statistical analysis

Descriptive statistics were presented as proportions, means, standard deviations, or medians and interquartile ranges. Categorical variables were compared using the chi-square test, and continuous variables were compared using the Student's t-test. Multivariate analysis was performed by constructing a logistic regression model, where the dependent variable was a failure to improve MR by at least two grades compared to the initial echocardiography. The explanatory variable was the return to sinus rhythm, adjusted for demographic and clinical characteristics that may act as mediators or confounders. A separate analysis was conducted on the group that returned to sinus rhythm, comparing characteristics across various measures within this group and those who remained in AF. A comparison of echocardiographic indices between the initial and follow-up tests was performed using the unpaired t-test. All statistical tests were two-sided, and a p-value of <0.05 was considered significant. Statistical processing was conducted using the IBM SPSS Statistics for Windows, Version 23.0. (IBM Corp., Armonk, NY).

## Results

Description of the study population

The study included 417 patients who underwent echocardiography during AF and were diagnosed with MR. Of these patients, 46% were men (n = 192), and the mean age was 74.6 ± 12 years. All patients had a follow-up echocardiography within six months. Table 1 displays the clinical and demographic characteristics of the participants, including data on their return to sinus rhythm. Hypertension was present in 62% of patients, diabetes in 28%, hyperlipidemia in 29%, and smoking in 16%. The mean age of the 254 patients (61%) who returned to sinus rhythm was lower than that of those who remained in AF (73.4 ± 12 vs. 76.4 ± 11 years, p = 0.016). The proportion of men in the sinus rhythm group was 49.6% (n = 126) compared to 40.5% of women (n = 66; p = 0.068). There was no significant difference in the rate of risk factors between the sinus rhythm group and those who remained in AF.

**Table 1:** Characteristics of the study population NA: not applicable; SD: standard deviation. All statistical tests were two-sided and a p-value of <0.05 was considered significant.

Demographic and clinical data	All (n=417)	Sinus (n=254; 61%)	Atrial fibrillation (n=163; 39%)	P-value
Mean age ± SD, years	74.6 ± 12	73.4 ± 12	76.4 ± 11	0.016
Male sex, n (%)	192 (46%)	126 (49.6%)	66 (40.5%)	0.068
Female sex, n (%)	225 (53.9%)	128 (50.3%)	97 (59.5%)	
Risk factors, n (%)	315 (75.5%)	194 (61.6%)	121 (38.4%)	NA
Hypertension, n (%)	197 (62.5%)	124 (63.9%)	73 (60.3%)	0.552
Diabetes, n (%)	89 (28.3%)	59 (30.4%)	30 (24.8%)	0.281
Hyperlipidemia, n (%)	93 (29.5%)	61 (31.4%)	32 (26.4%)	0.344
Smoker, n (%)	50 (15.9%)	32 (16.5%)	18 (14.9%)	0.702

Improvement in the severity of mitral regurgitation

Of the 417 patients, 100 (24%) showed improvement in MR by two or more grades on the follow-up echocardiography within six months (Table 2). There was no significant difference in the average age and sex of patients who improved by two or more grades compared to those with less improvement. Additionally, there was no association between risk factors and MR severity improvement. However, 72 of the 100 patients who improved by two or more grades had returned to sinus rhythm (28.4%). A higher proportion of patients who underwent cardioversion to sinus rhythm showed improvement in MR severity (182 patients, 57%; p = 0.0090).

**Table 2:** Improvement in mitral regurgitation (MR) severity and association with sinus rhythm and cardioversion *According to the ASE classification ASE: American Society of Echocardiography; MR: mitral regurgitation; Afib: atrial fibrillation

Improvement	All (n=417)	Normal sinus rhythm(n=93; 23%)	Abnormal rhythm - atrial fibrillation (n=324; 77%)	P-value
MR ≥ 2 degrees*, n (%)	100 (24%)	28 (30%)	72 (22%)	0.116
MR < 2 degrees, n (%)	317 (76%)	65 (70%)	252 (78%)	

Multivariate analysis logistic model

A multivariate logistic regression model was used to evaluate the relationship between conversion to sinus rhythm and improvement in MR severity. The model was adjusted for patient age, sex (reference group: women), and heart rate at the second test (reference group: AF). The results demonstrated a positive, independent, and statistically significant association between conversion to sinus rhythm and improvement in MR severity, with an odds ratio (OR) of 1.86 and a 95% confidence interval (CI) of 1.12 to 3.06 (p = 0.01).

Comparison of echocardiography measurements

A total of 254 patients were in sinus rhythm during the second echocardiography. Table 3 shows this group's clinical and demographic characteristics, categorized by the extent of improvement in MR. The mean age of these patients was 73.5 ± 12 years, with an equal distribution between the sexes. There was no significant difference in clinical and demographic data between patients who improved by two or more grades in MR severity and those who did not.

**Table 3:** Characteristics of study population by extent of improvement in MR severity NA: not applicable; MR: mitral regurgitation; SD: standard deviation Only 254 patients were included because they were in sinus rhythm. All statistical tests were two-sided, and a p-value of <0.05 was considered significant.

Demographic and clinical data	All (n=254)	MR<2 degrees (n=182; 72%)	MR≥2 degrees (n=72; 28%)	P-value
Mean age ± SD, years	73.5±12.6	73.3±12.6	73.9±12.6	0.711
Male sex, n (%)	194 (76%)	86(47.3%)	40 (55.6%)	0.233
Female sex, n (%)	60 (23.6%)	96 (52.7%)	32 (44.4%)	0.16
Risk factors, n (%)	194(76%)	141(72.7%)	53 (27.3%)	NA
Hypertension, n (%)	124(64%)	89 (63%)	35 (66%)	0.706
Diabetes, n (%)	59 (30%)	44 (31%)	15 (28%)	0.695
Hyperlipidemia, n (%)	61(31%)	45 (32%)	37 (30%)	0.818
Smoker, n (%)	32(16%)	22(15%)	10(19%)	0.585

Table 4 presents echocardiography data divided between patients who improved by two or more grades in MR severity and those who did not. Patients who showed significant improvement in MR severity after cardioversion to sinus rhythm were also associated with a significant reduction in left ventricular dimensions, left atrial dimensions, and transvalvular gradient. Additionally, the mitral valve morphology was normal in these patients.

**Table 4:** Comparison of echocardiography indices in AF versus improvement in MR severity AF: atrial fibrillation; MR: mitral regurgitation; LVIDd: left ventricular internal diameter end diastole; LVIDs: left ventricular internal diameter end systole; LVPWd: left ventricular posterior wall end diastole; LVPWs: left ventricular posterior wall end systole; IVSd: interventricular septal end diastole; IVSs: interventricular septal end systole; FS: fractional shortening; RV/IVS/PW: right ventricle/interventricular septum/posterior wall; RVDd: right ventricular end-diastolic diameter; Aod: aortic root diameter; Ascd: ascending aorta diameter; HR: heart rate; LA: left atrium; TI, tricuspid insufficiency; SD: standard deviation All statistical tests were two-sided, and a p-value of <0.05 was considered significant.

Echocardiography index	MR difference<2 (n: 317 (76%)			MR difference≥2 (n= 100 (24%)		
	AF, Mean ± SD (n= 252 (78%)	SINUS, Mean ± SD (n= 65 (70%)	P-Value	AF, Mean ± SD (n= 72 (22%))	SINUS, Mean ± SD (n= 28 (30%)	P-value
LVIDd (cm)	5.10 ± 0.77	5.19 ± 0.79	0.02	5.24 ± 0.82	5.21 ± 0.82	0.74
LVIDs (cm)	3.57 ± 0.96	3.57 ± 0.96	0.32	3.79 ± 0.99	3.58 ± 0.91	0.01
LVPWd (cm)	1.04 ± 0.25	1.05 ± 0.23	0.63	0.97 ± 0.21	1.01 ± 0.18	0.20
LVPWs (cm)	1.55 ± 0.37	1.58 ± 0.37	0.33	1.53 ± 0.39	1.51 ± 0.34	0.75
IVSd (cm)	1.28 ± 0.31	1.28 ± 0.31	0.97	1.24 ± 0.32	1.25 ± 0.24	0.80
IVSs (cm)	1.62 ± 0.35	1.65 ± 0.34	0.21	1.52 ± 0.32	1.60 ± 0.29	0.02
FS (%)	30.72 ± 10.48	32.60 ± 9.49	0.01	28.70 ± 10.17	32.17 ± 9.28	0.01
RV/IVS/PW	1.27 ± 0.32	1.26 ± 0.35	0.73	1.28 ± 0.38	1.25 ± 0.28	0.67
RVIDd (cm)	2.41 ± 0.54	2.51 ± 0.63	0.06	2.46 ± 0.61	2.48 ± 0.58	0.82
LA short axis (cm)	4.98 ± 0.71	5.02 ± 0.72	0.22	4.97 ± 0.64	4.79 ± 0.76	0.02
Aod (cm)	3.36 ± 0.52	3.38 ± 0.50	0.62	3.34 ± 0.47	3.42 ± 0.47	0.10
ASCd (cm)	1.81 ± 0.45	1.82 ± 0.34	0.75	1.81 ± 0.33	1.92 ± 0.36	0.03
HR	91.5 ± 28.1	72.6 ± 15.7	<0.01	87.3 ± 26.8	68.6 ± 14.8	<0.01
LA area (cm^2^)	27.60 ± 6.0	26.01 ± 5.8	<0.01	27.31 ± 6.0	26.09 ± 6.7	0.02
LA volume (mL)	112.3 ± 45.2	104.0 ± 37.4	0.06	109.2 ± 37.4	102.4 ± 37.5	0.24
Jet area (cm^2^)	10.29 ± 4.5	10.06 ± 4.6	0.57	11.37 ± 3.7	5.9 ± 1.7	<0.01
Vena contracta (cm)	0.68 ± 0.17	0.60 ± 0.15	<0.01	0.66 ± 0.08	0.56 ± 0.07	<0.01
TI gradient (mmHg)	39.32 ± 13.8	40.84 ± 15.2	0.10	41.85 ± 12.9	36.5 ± 10.7	<0.01

The study examined echocardiography parameters in patients who maintained sinus rhythm at six months with improvement in MR and those who maintained sinus rhythm but did not improve in MR also evaluated patients with AF at baseline with MR improvement at six months and patients with AF at baseline and at six months without MR improvement.

The multivariate analysis revealed a significant association between sinus rhythm restoration and MR severity improvement by two or more grades (p = 0.01, OR = 1.9, 95% CI (1.2 to 3.0)). The study also demonstrated improvements in other echocardiography parameters. Specifically, there was a decrease in the end-systolic left ventricular diameter from 3.79 ± 0.99 to 3.58 ± 0.91 cm (p = 0.01), a reduction in left atrial diameter from 4.97 ± 0.64 to 4.79 ± 0.76 cm (p = 0.02), a decrease in jet area from 11.37 ± 3.7 to 5.9 ± 1.7 cm² (p < 0.01), a reduction in vena contracta from 0.66 ± 0.08 to 0.56 ± 0.07 cm (p < 0.01), and a decrease in tricuspid insufficiency gradient from 41.85 ± 12.9 to 36.5 ± 10.7 mmHg (p < 0.01). Furthermore, the fractional shortening index improved from 28.70 ± 10.17% to 32.17 ± 9.28% (p = 0.01).

Conversely, patients with minimal or no improvement in MR exhibited an increase in end-diastolic diameter from 5.10 ± 0.77 to 5.19 ± 0.79 cm (p = 0.02) and an increase in left atrial diameter. Patients with AF at baseline and six months who showed improvement in MR had significant reductions in left ventricular dimensions, left atrial dimensions, and tricuspid regurgitation velocity, indicating improved cardiac function. In contrast, patients with AF at baseline and six months without improvement in MR showed significant reductions in left ventricular dimensions and tricuspid regurgitation velocity but no significant improvement in left atrial dimensions.

## Discussion

This study explored the relationship between AF and functional MR, demonstrating that cardioversion to sinus rhythm is a sensitive predictor of MR improvement. Patients who returned to sinus rhythm showed improved left ventricular dimensions, left ventricular function, and MR severity. These findings suggest that AF may contribute to worsening MR rather than merely coincidental.

Previous studies have investigated the association between AF and secondary MR, but none have established a causal connection [11,12]. Gertz et al. examined patients undergoing AF ablation with at least moderate MR and compared them with a control group without MR. They found that patients who maintained sinus rhythm experienced improvements in mitral ring dimensions, left atrium size, and MR severity, suggesting that functional MR may be caused by mitral ring expansion and left atrial dilation [1]. Other studies have shown that AF-induced left atrial dilation leads to significant mitral ring expansion [11,13,14].

In contrast, Zhou et al. found that AF causes more dilation of the tricuspid annulus than the mitral annulus, leading to moderate or greater tricuspid regurgitation in a significant proportion of patients. They attributed the lower incidence of significant MR to the more robust fibrous skeleton of the mitral annulus than the tricuspid annulus. While these findings do not rule out the possibility that AF and annular dilation can cause significant MR, they suggest that this type of MR may be less prevalent than others [13].

Further work with three-dimensional echocardiography has enhanced our understanding of mitral annulus structure and function in patients with MR [14]. However, the prognostic significance of functional MR remains unclear. Mitral regurgitation accompanied by ventricular dysfunction worsens prognosis compared to cardiomyopathy with normal mitral valve function and is associated with higher mortality in organic MR, even in asymptomatic patients [15-17]. Additionally, left atrial dilation in patients with AF alone is an independent predictor of morbidity and mortality [18].

This study was limited as it is a retrospective analysis. Therefore, it only included data for up to six months and cannot determine long-term outcomes. Despite this limitation, our findings offer important insights into the relationship between AF and functional MR and may inform treatment decisions for patients with these conditions. Further research is needed to investigate the role of rhythm control in patients with functional MR.

## Conclusions

Our study suggests that restoring sinus rhythm can notably enhance functional MR, potentially minimizing the need for surgical intervention. Ideally, treatment decisions should be made when the patient is in sinus rhythm. However, further large-scale prospective studies are required to assess whether controlling rhythm or rate is more effective in managing functional MR. These findings indicate that patients with AF and significant MR might benefit from sinus rhythm restoration, which could lead to better cardiac function and overall outcomes.
